# Medical Students, Their Schools and Schoolmasters

**Published:** 1886-11-06

**Authors:** 


### MEDICAL STUDENTS, THEIR SCHOOLS AND SCHOOLMASTERS.

*The Student Father to the Doctor.* THE welfare of medical students is a matter of much concern to themselves and their parents, and of some interest to the outside public. Medical men play so important a part in our social system, and their relations with others are of such an intimate and personal nature, that their antecedents and character can never be matters of indifference. If it be generally true that "the child is father to the man," it is still more certain that the student is father to the practitioner. As is the one in attainments, conduct, and bearing, so in all probability will be the other. There is not only a relation of continuity and succession, but also of cause and effect. That being so, it will be time well spent to take a brief survey of some considerations appertaining to medical students as a class.

*Where do they come from?* It is not uninteresting to ask "Whence come they?" For the most part they come from the great middle-class, which novelists of superior tastes, acquired, no doubt, from an extensive and familiar knowledge of the aristocracy, so affect to patronise and sneer at; a class, however, which consists mainly of persons of understanding and solid character. Coming from such a class, our medical stndents, as a whole, begin the race of life with inherited qualities and acquired habits of sobriety, sense, and virtue. On this foundation of birth and habit the superstructures of medical education and personal character are to be reared by such architects and builders as the circumstances of the case will afford.

*Five Years without a Home.* His preliminary examination passed and registration completed, the student is fairly launched on his career. For the next four or five years the hospital is his school, but where is his home? Has he a home at all? In too many cases it is to be feared he has not. And yet he is but a youth—a youth with wilful passions and small experience. A sigh of pity may well be spared for him if he sometimes fails to walk in the ways which a severe prudence would consider wise and good. "How oft the sight of means to do ill deeds makes ill deeds done." The wise words of Shakespeare's experience readily occur to the mind as it thinks of the innumerable temptations of a student's life, and the few and feeble restraints which are available for check and guidance to him. The solitary lodging, the ill-cooked food, the frowsy attendant, the stuffy street—these are the unsavoury surroundings which the youth has exchanged for the country vicarage or the pleasant farm. The dry bones, the tedious muscles, with their origins and insertions a thousand times more tedious still, often seem the very invention of Satan for the torment of youthful anatomists. Who can wonder if the strong-limbed Hercules, with his bounding pulses, sometimes consigns them all to prompt perdition, and seeks his pleasure in the billiard-room, or the theatre, or some place more dangerous still?

*What do his Teachers do for him?* What does the hospital do for the youth? What do his teachers do for him? In some cases the least they can. They are very anxious to receive his "composition fees." One would be pleased to see them a little more anxious to render him some "compensation services." No one doubts that the hospitals and their teaching members are hard pressed by the competition of the times, and probably the remuneration of teachers is in many cases painfully small. Still, it is not pleasant to think that because of this, fresh and innocent youths from the country and elsewhere are tossed into the whirlpool of London temptations with scarcely a hand held out for their succour, or even an eye to see their danger. Youth and discipline should be inseparable if manhood and virtue are to be united. In none of the hospitals is there any adequate discipline, and in some there is practically none at all.

*The Collegiate System.* There is perhaps no immediately available remedy for these things, but it is incumbent upon hospital authorities and teachers (the former as much as the latter) to make some decided effort in the direction of discipline and guidance. The Collegiate system, or some modification of it, demands at least a consideration. At some hospitals it already exists in a tentative kind of way. If it be found to work well, steps should be taken for its extension and improvement. In any case, if young men fail to reach a high average of technical skill and personal merit, the blame must not be placed entirely on their own shoulders. It is impossible to absolve the system of education and its managers from a large portion of the responsibility. In another column we have said something about residential accommodation for the students, and we propose to revert to the subject before long.

